# Effects of acute aerobic exercise on resting state functional connectivity of motor cortex in college students

**DOI:** 10.1038/s41598-024-63140-6

**Published:** 2024-06-27

**Authors:** Wenyi Li, Bingyang Wang, Haoteng Yuan, Jun Chen, Gonghe Chen, Yue Wang, Shilin Wen

**Affiliations:** 1 Department of Physical Education and Training, Capital University of Physical Education and Sports, Beijing, 100191 China; 2 Department of Ideological, Political and General Education, Guangzhou Huashang Vocational College, Jiangmen, 529152 Guangdong China; 3 Department of Physical Education, Changsha Medical University, Changsha, 410000 Hunan China; 4 Department of Physical Education, North China Institute of Aerospace Engineering, Langfang, 065000 Hebei China

**Keywords:** Acute aerobic exercise, Resting state functional connectivity, Motor cortex, Exercise intensity, Fitness level, Health care, Health policy, Health services, Public health

## Abstract

This study intends to inspect the effects of acute aerobic exercise (AE) on resting state functional connectivity (RSFC) in motor cortex of college students and the moderating effect of fitness level. Methods: 20 high fitness level college students and 20 ordinary college students were recruited in public. Subjects completed 25 min of moderate- and high-intensity acute aerobic exercise respectively by a bicycle ergometer, and the motor cortex’s blood oxygen signals in resting state were monitored by functional Near Infrared Spectroscopy (fNIRS, the Shimadzu portable Light NIRS, Japan) in pre- and post-test. Results: At the moderate intensity level, the total mean value of RSFC pre- and post-test was significantly different in the high fitness level group (pre-test 0.62 ± 0.18, post-test 0.51 ± 0.17, *t*_(19)_ = 2.61, *p* = 0.02, *d* = 0.58), but no significant change was found in the low fitness level group. At the high-intensity level, there was no significant difference in the difference of total RSFC between pre- and post-test in the high and low fitness group. According to and change trend of 190 “edges”: at the moderate-intensity level, the number of difference edges in the high fitness group (*d* = 0.58, 23) were significantly higher than those in the low fitness group (*d* = 0.32, 15), while at high-intensity level, there was a reverse trend between the high fitness group (*d* = 0.25, 18) and the low fitness group (*d* = 0.39, 23). Conclusions: moderate-intensity AE can cause significant changes of RSFC in the motor cortex of college students with high fitness, while high fitness has a moderating effect on the relationship between exercise intensity and RSFC. RSFC of people with high fitness is more likely to be affected by AE and show a wider range of changes.

## Introduction

Physical activity is a non-pharmacological strategy to improve physical and mental health throughout the life cycle. This strategy can not only induce the adaptive changes of human immune system^1^ and improving the functions of respiratory system^2^ and cardiovascular systems^3^. It can also promote brain cognition, including motor skill learning^4,5^, emotion regulation^6^, and social interaction^7^. The reason why physical activity can promote brain cognition is that the human body has a very strong ability of stress and adaptation. Once exposed to the external environment rich in stimulation, the brain neurons may undergo plasticity changes adapted to the environment^8–10^. The evidence for research on physical activity for brain health is likely to span disciplines as diverse as exercise science, biochemistry, and cognitive neuroscience. It can be seen that the topic of physical activity promoting brain health is a highly interdisciplinary research issue in various disciplines. At the same time, exploring what kind of exercise can bring the best exercise benefits and the corresponding neural mechanism is also a research hotspot in the field of sports science.

Aerobic exercise is a recognized economic, green and convenient way of physical activity^11^, which has a positive impact on the improvement of mental health level, the improvement of cognitive decline, and the regulation of negative emotions. Aerobic exercise mainly includes two types: long-term aerobic exercise and acute aerobic exercise (AE). The cognitive and brain health benefits of long-term aerobic exercise are indisputable, but some unrelated factors (such as social activities, sleep, diet, etc.) may work together to affect the plasticity of the human brain, and the effects of these unrelated factors are difficult to manipulate and isolate during the experiment, and may mask the processing effects of long-term aerobic exercise. This makes it difficult to interpret the results. However, AE has many unique advantages and has been favored by researchers, such as exercise intensity, exercise time, exercise form and other variables are easy to manipulate in the experiment. Some studies have shown that AE can effectively enhance the plasticity of neurons, and the subjects’ emotional^12^, memory^13^ and attention^14^ functions can be temporarily improved (such as the efficacy is maintained within 1 h), which indicates that acute aerobic exercise can sensitively and temporarily stimulate adaptive changes in human brain neurons. At the same time, some studies have also pointed out that the exercise benefits obtained from long-term regular aerobic exercise may be the result of the cumulative effect produced by several acute exercises^15^. So, exploring the motor effects of acute exercise is helpful to understand the laws of cognitive or brain plasticity changes caused by long-term exercise training adaptation.

There is a specific relationship between AE and motor effects and there are two dominant hypotheses: the arousal theory hypothesis (also known as the inverted *U*-shaped hypothesis) and the drive theory hypothesis. Arousal theory hypothesis^16^ holds that the relationship between exercise intensity and exercise effect is not linear, but presents an inverted *U*-shaped relationship, that is, the most appropriate arousal level can be induced when moderate intensity exercise load is adopted, and the best exercise effect can be obtained correspondingly. The drive theory^15^ argues that the relationship between exercise intensity and motion effect may be linear, that is, high exercise intensity corresponds to the maximum motion effect. Nevertheless, the two theories have one thing in common: compared with moderate and high intensity exercise loads, low-intensity exercise causes the least cognitive and brain health effects. It can be seen that exercise intensity may be an important regulating variable affecting the exercise effect of AE, and the relationship between them has two different changing trends. However, in healthy college students with different fitness level, whether there is a different relationship between exercise intensity and exercise effect needs to be proved by empirical research.

With the development of neuroimaging techniques and the human connectome^17^ in the field of neuroscience, a new methodological basis has been provided for the evaluation of motor effects and the explanation of related neural mechanisms. There are widely distributed low-frequency fluctuations in human brain that can reflect certain key functions of the brain^18,19^. Some researchers believe that: The neural mechanism of the motor effect induced by physical activity may be related to the “change in the temporal coherence of spontaneous low-frequency fluctuations between neurons in different brain regions after exercise”^20^. However, the temporal correlation of “low-frequency fluctuations related to function” is called “resting state functional connectivity (RSFC)”. Because of its intuitive principle and simple calculation, RSFC is applied in the field of sports science as a macro index to evaluate the characteristics of low-frequency fluctuations in the resting state of the brain. Some studies have shown that with age, RSFC decreases in tandem with brain structural degradation and cognitive decline^21,22^. Results from another intervention study showed that: resveratrol supplementation in older adults enhanced memory function and was associated with enhanced RSFC in the medial hippocampus and between the left posterior hippocampus and medial prefrontal cortex^23^. They also found that the magnitude of RSFC enhancement in older adults correlated with the degree of memory improvement and the trend of glucose metabolism improvement^23^. From the above research evidence, RSFC is a relatively stable biomarker that can be used to characterize brain functional networks and explain changes in brain networks induced by physical activity. At the same time, considering the relationship between fitness level and RSFC, some studies have shown that fitness level (including skill level, skill learning ability, etc.) is correlated with RSFC of local brain regions, and RSFC of different fitness levels may also be different^24,25^. Therefore, fitness level may be a potential regulator of the effects of AE on RSFC. Exploring the effects of AE on RSFC in people with different fitness levels is helpful to understand which exercise intensity AE can obtain the greatest exercise benefits for different people, providing a theoretical reference.

In the process of physical activity, the brain areas related to sensory processing and the cerebral cortex related to motor control (such as the primary motor cortex and auxiliary motor area) may be the regions most susceptible to external stimuli and plastic changes^26^. Therefore, exploring the changes in the motor cortex RSFC before and after exercise will help to understand the changes in the resting network of the human brain that correspond to the regulation of brain resources during the preparation, initiation or execution of motor control. Based on this, our study intends to use the motor cortex as an area of interest to explore the effects of AE on RSFC.

In conclusion, in order to clarify which intensity can bring the best exercise benefits, and how to design aerobic exercise programs for people with different fitness levels, it is necessary to explore the mode of action of AE on RSFC and the corresponding neural mechanism. Therefore, the following hypotheses were proposed: (1) Different AE intensities would have differential effects on motor cortex RSFC of college students; (2) Fitness level has a regulating effect on the processing effect of AE.

## Materials and methods

### Participants

Twenty college athletes from a sports university were recruited into the high fitness group (H Group), and twenty ordinary college students from another university were recruited into the low fitness group (L Group)^27^. The laboratory assistant explained the basic protocol of the study to the proposed individuals by telephone or E-mail, and individuals interested in the study were included in the groups. Inclusion criteria: (1) normal intelligence, no history of brain injury or psychiatric disorders; (2) no symptoms of cold or flu, no major mood swings, etc. during the experiment; (3) right-handed; (4) no alcohol consumption,no late nights, no heavy exercise or physical work in the 3 days before the experiment; (5) participants in the high fitness group were required to hold a certificate of National Sports Skill Level-2 or above issued by the Sports Bureau and engage in at least 2 years of exercise training. Before the experiment, the assistant collected the basic information of all participants (see Table 1).

**Table 1:** Demographic data.

Variables	H Group (n = 20)	L Group (n = 20)	*df*	*t*-value	*U*-value	*Z*-value	p-value
Gender	Male	Male	–	–	–	–	–
Age (years)	20.00 (19.25, 20.00)	20.00 (19.25, 22.75)	–	–	145.00	− 1.59	0.14
Education (years)	14.00 (14.00, 14.00)	14.50 (14.00, 15.75)	–	–	139.50	− 1.93	0.10
Height (cm)	180.00 ± 6.76	176.60 ± 5.20	38	1.8	–	–	0.08
Weight (kg)	70.85 ± 7.65	71.60 ± 8.08	38	− 0.30	–	–	0.77
BMI (kg/m^2^)	21.83 ± 1.71	22.92 ± 1.92	38	− 1.89	–	–	0.07

H Group, high fitness group; L Group, low fitness group; BMI, body mass index; Normally distributed measurement data are described as Mean (± SD) and non-normally distributed measurement data are described as Me (Q1, Q3). Mean: arithmetic mean; SD: standard deviation; Me: median; Q1: the first quartile; Q3: the third quartile.

### Ethics statement

The studies involving human participants were reviewed and approved by Capital University of Physical Education and Sports Ethical Committee (Approval ID 2020A07). All methods are carried out in accordance with the relevant guidelines and regulations. The participants provided their written informed consent to participate in this study. Written informed consent was obtained from the individual(s) for the publication of any potentially identifiable images or data included in this article.

### Experimental procedures

In this study, fitness level (high fitness level group and low fitness level group) were taken as inter-subject variables, load intensity (moderate intensity and high intensity) and test order (pre-test and post-test) were taken as intra-subject variables, and RSFC of the motor cortex of the subjects’ brain was taken as dependent variable. The effects of different exercise load intensity before and after intervention on RSFC of the motor cortex of college students at different fitness levels were analyzed. All subjects completed two independent acute aerobic exercise experiments in different experimental periods (load intensity: moderate intensity and high intensity, and the interval between the two experimental periods was 72 h^28^). The two experimental conditions were counterbalanced across participants.The timing of the experimental sessions was controlled between 10 a.m. and 3 p.m., to exclude the influence of biorhythm on the results. Significantly, We conducted daily telephone communication with the subjects during the first three days of the experiment to remind them to avoid drinking, staying up late and high-intensity exercise.

**Figure 1:**
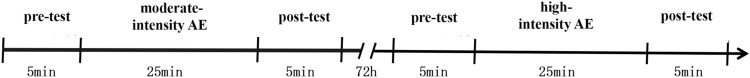
General experimental procedure.

In each experimental period, the participants first rested in the waiting room for 10 min and then completed the fNIRS pre-test, acute aerobic exercise and fNIRS post-test in the laboratory, as shown in Fig. 1. In the moderate-intensity segment, the participants first completed 25 min of aerobic exercise at 60–69% HRmax intensity, after which the participants’ HR returned to basal heart rate ± 10%^29^ (approximately 15–20 min after aerobic exercise) before performing the fNIRS test to avoid attenuation of exercise effects caused by too long or too short a rest period. Compared with the moderate-intensity section, in the high-intensity segment, all subjects were asked to complete 25 min of aerobic exercise at 80–89% HRmax intensity.

### Acute aerobic exercise intervention

Based on the classification standard of aerobic exercise intensity for healthy adults by the American College of Sports Medicine^30^, 60–69% and 80–89% HRmax were used as target HR ranges for exercise at moderate- and high-intensity intervals for the participants, respectively. Each participant asked to complete the AE program on a power bike (ergoselect 100 K, German origin, resistance range 20–999 w) and wear a Nissan Polar HR monitor during exercise. All participants completed the AE program for 25 min, including a 5-min warm-up, a 15-min bout of exercise at target HR, and a 5-min cool-down. A total of 8 HR indicators were collected during exercise: 7, 9, 11, 13, 15, 17, 19 and 20 min, respectively (after warming up, HR was recorded every 2 min).The rating of perceived exertion(RPE) was used to evaluate the effort of the participants during the experiment^31^. And the RPE values were recorded immediately after the start of the fNIRS pre-test, immediately after the exercise, and immediately after the fNIRS post-test. If participants showed signs of distress (pale complexion or lip color) or abnormal physiological signals (abnormal HR) during acute aerobic exercise, the trial was terminated immediately.

### fNIRS measurement

**Figure 2:**
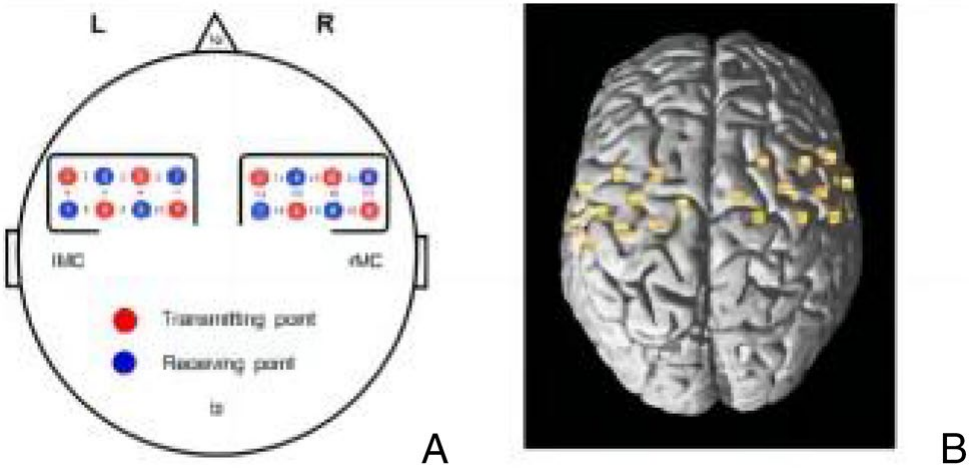
Measurement channel configuration according to international 10/20 system and MNI spatial registration. *Note*: (**A**) fNIRS measurement channel spatial distribution; (**B**) MNI spatial registration.

Functional near infrared spectroscopic image (fNIRS, Nissan Shimadzu portable LightNIRS) was used to monitor resting-state cerebral blood oxygen data from participants, with 20 source-detector pairs (8 light emitter and 8 detectors) positioned according to the international 10/20 system (corresponding to 20 measurement channels, see Fig. 2), with a sampling frequency of 10 Hz and 3 wavelengths of near-infrared light (780 nm, 805 nm, 830 nm). The interoptode distance was 3 cm. The fNIRS topographic map covered the motor cortex. Using fNIRS to measure RSFC in the motor cortex helps to support RSFC changes in the motor cortex based on fNIRS data and predict the effect of exercise training. The exact position of the photodetector on the scalp was measured using a 3D localizer, NIRS_SPM was used to confirm the registration of the standard brain template and 3D location, and then the MNI coordinates of each channel and the corresponding brain region of the channel were obtained, see Table 2. After the cap was worn, the photodetector was checked and adjusted to ensure that all participants were wearing the same position. The experiments were conducted in the Cardiorespiratory Function Laboratory of the Capital University of Sports and Physical Education. All participants completed a 5-min fNIRS resting-state scan before and after aerobic exercise, and were asked to close their eyes but not to fall asleep.

**Table 2:** MNI coordinates and corresponding anatomical positions of fNIRS channels.

Channels	MNI			Region
	X	Y	Z	
1	− 59	− 2	43	Premotor and supplementary motor cortex_L
2	− 44	6	58	Premotor and supplementary motor cortex_L
3	− 27	6	69	Premotor and supplementary motor cortex_L
4	− 65	− 19	41	Primary sensory-motor cortex_L
5	− 51	− 11	57	Primary motor cortex_L
6	− 35	− 7	67	Premotor and supplementary motor cortex_L
7	− 14	− 8	77	Premotor and supplementary motor cortex_L
8	− 57	− 26	55	Primary sensory-motor cortex_L
9	− 42	− 20	68	Primary motor cortex_L
10	− 24	− 18	76	Premotor and supplementary motor cortex_L
11	25	12	69	Premotor and supplementary motor cortex_R
12	45	13	58	Dorsolateral prefrontal cortex_R
13	57	13	39	Opercular part_R
14	13	− 4	76	Premotor and supplementary motor cortex_R
15	34	− 1	66	Premotor and supplementary motor cortex_R
16	52	− 2	56	Premotor and supplementary motor cortex_R
17	64	2	37	Premotor and supplementary motor cortex_R
18	22	− 15	76	Premotor and supplementary motor cortex_R
19	42	− 14	69	Premotor and supplementary motor cortex_R
20	58	− 14	54	Primary sensory-motor cortex_R

L is the left hemisphere of the brain, R is the right hemisphere of the brain.

### fNIRS data processing and visualization

The collected raw data were imported into LightNIRS analysis system, and the HbO blood oxygen signal and light intensity signal were converted into TXT format and stored in the storage. FC-NIRS software package is used to preprocess the original data and calculate RSFC: (1) Preprocessing. In the preprocessing stage^32^, the raw optical intensity was first adjusted to generate a relative (percent) concentration change by dividing by the mean of the intensity, and then the Modified Beer-Lambert law was used to transform each channel’s light intensity data into the HbO concentration signal. FC-NIRS utilizes a band-pass filter with third-order Butterworth, zero-phase digital filtering for low-pass and fifth-order Butterworth, zero-phase digital filtering for high-pass to remove low-frequency noise, and physiological interference source. The filtering range for the band-pass filter could be defined by ourselves according to the study objectives. For our study, we used a default band-pass range from 0.01 to 0.1 Hz, which represents the frequency range of hemodynamic signals that are thought to emanate from spontaneous neural activity. The spline interpolation method and correlation-based signal improvement (CBSI) method are used to reduce the motion-induced artifacts. Previous studies have shown that the system signal increases or decreases over time due to long-term physiological changes and that motion-related noise remains. In fNIRS data preprocessing, linear trends are usually removed. FC-NIRS also estimated the linear trend with a least-square fit of a straight line and then subtracted it from the hemoglobin concentration signals^32^. (2) Quality control. The quality control module detects motion artifacts, calculates the signal-to-noise ratio (SNR), calibrates the bad conductance and eliminate the unqualified data. To detect the signal variations inside a series of sliding windows for the head motion check, FC-NIRS calculated the sliding standard deviation of the time series of concentration signals. The resulting time series of the sliding standard deviation was divided by a predetermined threshold value *T* and the values above the threshold value *T* were considered to be motion artifacts. (3) FC calculation. Two different methods—a whole-brain correlation method and a seed-based correlation method—are provided by FC-NIRS for the FC calculation. We applied whole-brain correlation analysis to calculate FC by calculating the strength of connectivity between any two measured channel pairs within the whole cerebral cortex. Pearson’s correlation coefficient *r* values between any two measurement channel pairs was calculated, and the Fisher-z transform was used to obtain the Z-value of the r-transformed Z-value as a representative indicator of the strength of the FC, and the Z-value matrix of the RSFC for all channels was obtained. Based on this, the RSFC values at the clusters level were further calculated. The BrainNet Viewer software package was applied to image the “edges” where there were differences between two groups (*p* < 0.05) (https://www.nitrc.org/projects/bnv/).

### Statistical analysis

The FC-NIRS software was applied to complete the RSFC calculations at the individual level and in clusters, and the experimental data were statistically analysed via the Matlab data platform and SPSS 22.0 software (SPSS Inc., Chicago, IL, United States). For demographic information, we used independent samples *t*-tests or non-parametric tests, and used a paired samples *t*-test to test the changes in RSFC of college students before and after moderate intensity and high intensity AE, as well as the changes in 180 “edges” (the correlation coefficient r value for each measured channel), the significance level set at *p* < 0.05, uncorrected.

## Results

### Process monitoring of acute aerobic exercise

**Figure 3:**
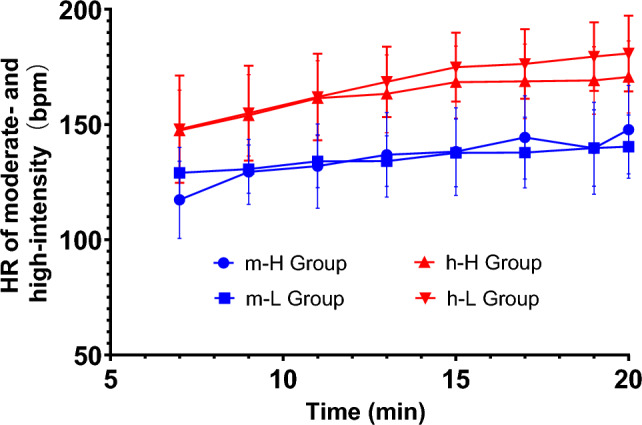
Heart rate Mean changes at 8 time points in the high and low fitness groups. *Note*: m-H Group for HR of high fitness group at moderate-intensity exercise, m-L Group for HR of low fitness group at moderate-intensity exercise, h–H Group for HR of high fitness group at high-intensity exercise, h–L Group for HR of low fitness group at high-intensity exercise.

During moderate-intensity AE, the HR of the high fitness group was 135.71 ± 9.53 beats per minute (bpm), equivalent to 67.84 ± 4.76% HRmax, and in the low fitness group was 135.43 ± 4.18 bpm, equivalent to 68.06 ± 2.10% HRmax, with both groups achieving the required target-HR value range of 60–69% HRmax. During the high-intensity AE, the HR of the high fitness group was 162.86 ± 8.32 bpm, equivalent to 80.97 ± 4.23% HRmax, while that of the low fitness group was 168.11 ± 12.08 bpm, equivalent to 83.48 ± 6.03% HRmax, with both groups achieving the target-HR range of 80–89% HRmax, see Fig. 3. The results of the paired samples *t*-test showed that there was a significant difference in HR between moderate- and high-intensity exercise in the high fitness group (*t*_(7)_ = − 24.48, *p* = 0.00); similarly, there was a significant difference in HR between moderate- and high- intensity exercise in the low fitness group (*t*_(7)_ = − 11.27, *p* = 0.00).

In addition, during moderate-intensity AE, the RPE values for high and low fitness groups were 11.95 ± 2.33 and 12.30 ± 1.34 respectively, both in the range 11–14, i.e. in the “light” and “somewhat hard” range. The RPE values measured before fNIRS (7.70 ± 1.78, 7.50 ± 1.05) and after fNIRS (8.10 ± 2.25, 7.65 ± 1.81) were in the range 7–10, i.e. in the “very light” and “very light” range. During the higher intensity AE, the RPE values in the high and low fitness groups were 14.50 ± 2.14 and 14.10 ± 2.47 respectively, both in the range 14–16, i.e. in the “somewhat hard” and “hard” range. The RPE values measured before fNIRS (7.20 ± 0.22, 7.40 ± 1.05) and after fNIRS (7.35 ± 1.64, 7.15 ± 0.93) were in the range 7–8, which is in the “very light” range for both groups.

### Effects of moderate-intensity AE on RSFC in the motor cortex of college students

**Figure 4:**
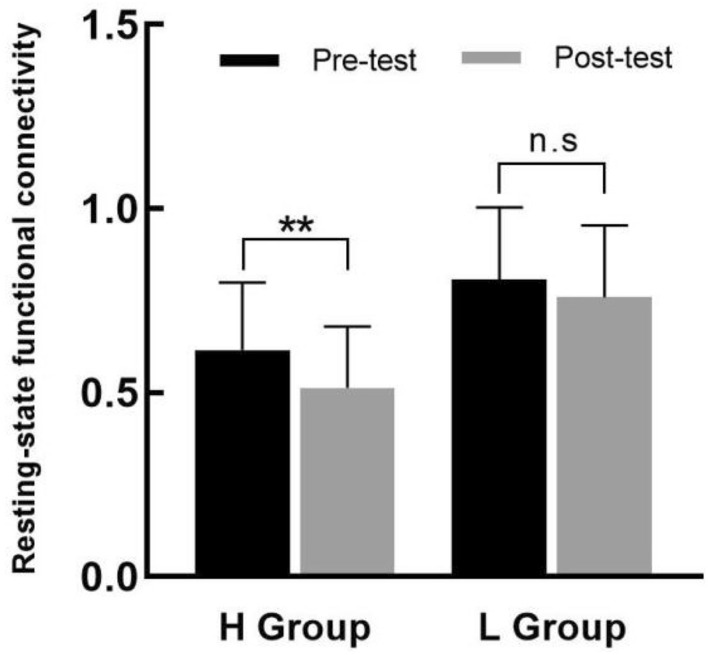
Comparison of total RSFC pre- and post-test of moderate-intensity AE exercise in high and low fitness groups. *Note*: ** for *p* < 0.05, n.s for *p* > 0.05.

The study further conducted a paired samples *t*-test for the total RSFC pre- and post-test for each of the high and low fitness groups separately(see Fig. 4). The results showed that there was a significant difference in the total RSFC pre- and post-test(pre-test: 0.62 ± 0.18, post-test: 0.51 ± 0.17, *t*_(19)_ = 2.61, *p* = *0*.02, *d* = 0.58) in the high fitness group, but not in the low fitness group[(pre-test: 0.81 ± 0.20, post-test: 0.76 ± 0.20, *t*_(19)_ = 1.13, *p* = 0.27 0.20, *d* = 0.32)]. The results showed that AE only had a significant effect on RSFC in the motor cortex of high fitness level college students at moderate-intensity and that effect size in the high fitness group was greater than in the low.

**Figure 5:**
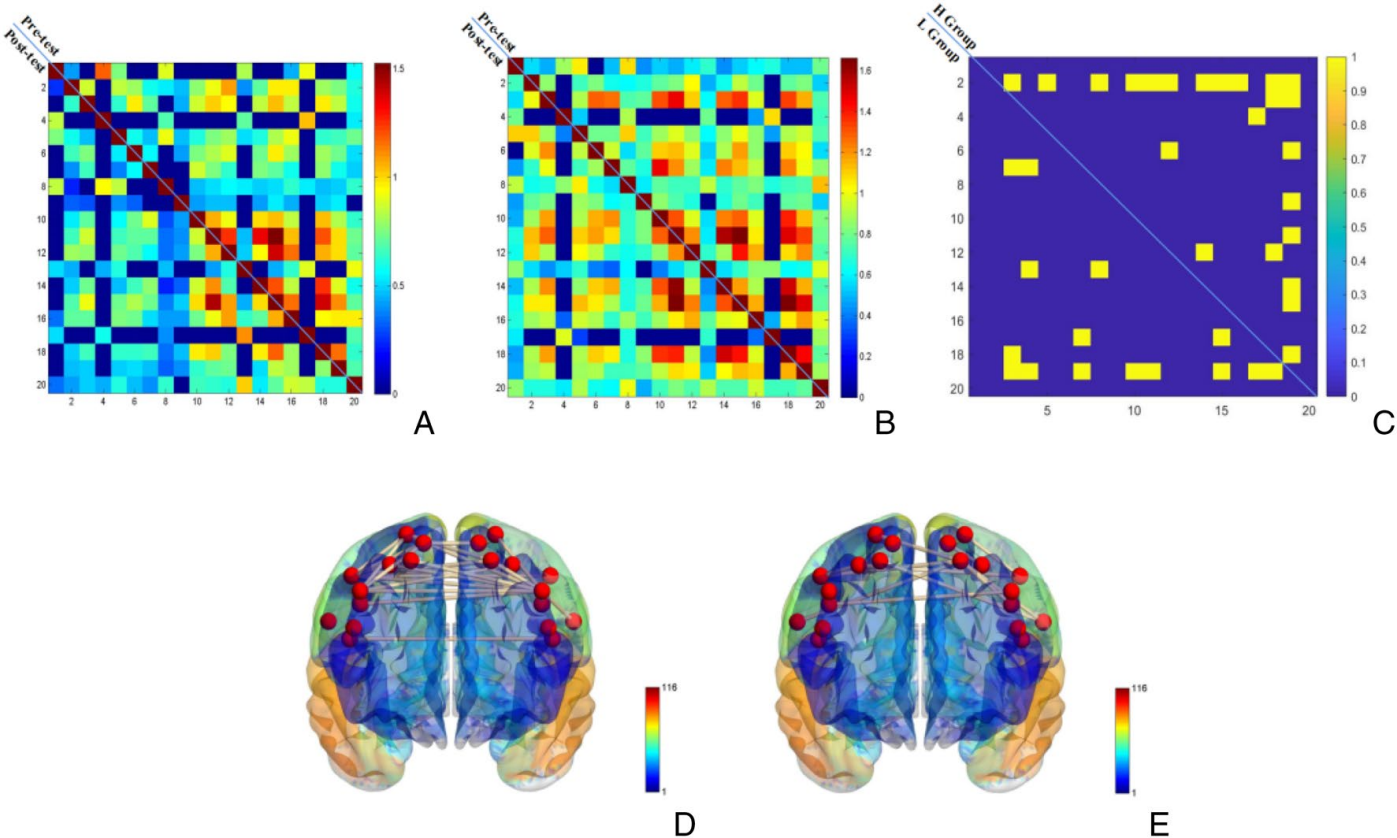
*p* value (p < 0.05, uncorrected) matrix map and brain network map of pre- and post-test of moderate-intensity AE in high and low fitness groups. *Note*: (**A**) and (**B**) show the RSFC matrices for the pre- and post-test for the high and low fitness groups, respectively, (**C**) shows the *p*-value (*p* < 0.05, uncorrected) matrices for the pre- and post-test for the high and low fitness groups, (**D**) and (**E**) show the brain networks with differential “edges” for the high and low fitness groups, respectively.

Paired samples *t*-tests were conducted on single edges of the RSFC pre- and post-test (n = 190) (see Fig. 5A, B, C), and the results showed that a total of 23 edges were significantly different pre- and post-test in the high fitness group, while a total of 15 edges in the low(see Fig. 5D, E). The results indicate that at moderate-intensity level, the RSFC in the high fitness group showed more sensitivity to AE stimulation compared to the low fitness group.

### Effects of high-intensity AE on RSFC in the motor cortex of college students

**Figure 6:**
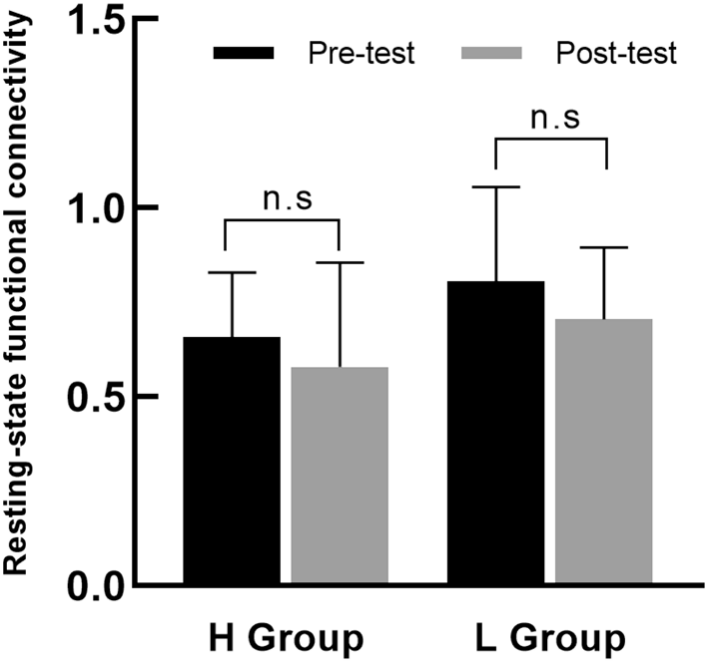
Comparison of total RSFC pre- and post-test of high-intensity AE exercise in high and low fitness groups. *Note*: ** for *p* < 0.05, n.s for *p* > 0.05.

The study further conducted a paired samples *t*-test for the total RSFC pre- and post-test for the two groups (see Fig. 6). The results showed that there was no significant difference in the total RSFC between pre- and post-test in the two groups (high fitness group: *t*_(19)_ = 1.42, *p* = 0.17, *d* = *0*.25; low fitness group: *t*_(19)_ = 1.75, *p* = 0.10, *d* = 0.39). The results indicated that AE have no significant effect on RSFC in the motor cortex of college students at both groups, but the effect size in the low fitness group was greater than in the high fitness group.

**Figure 7:**
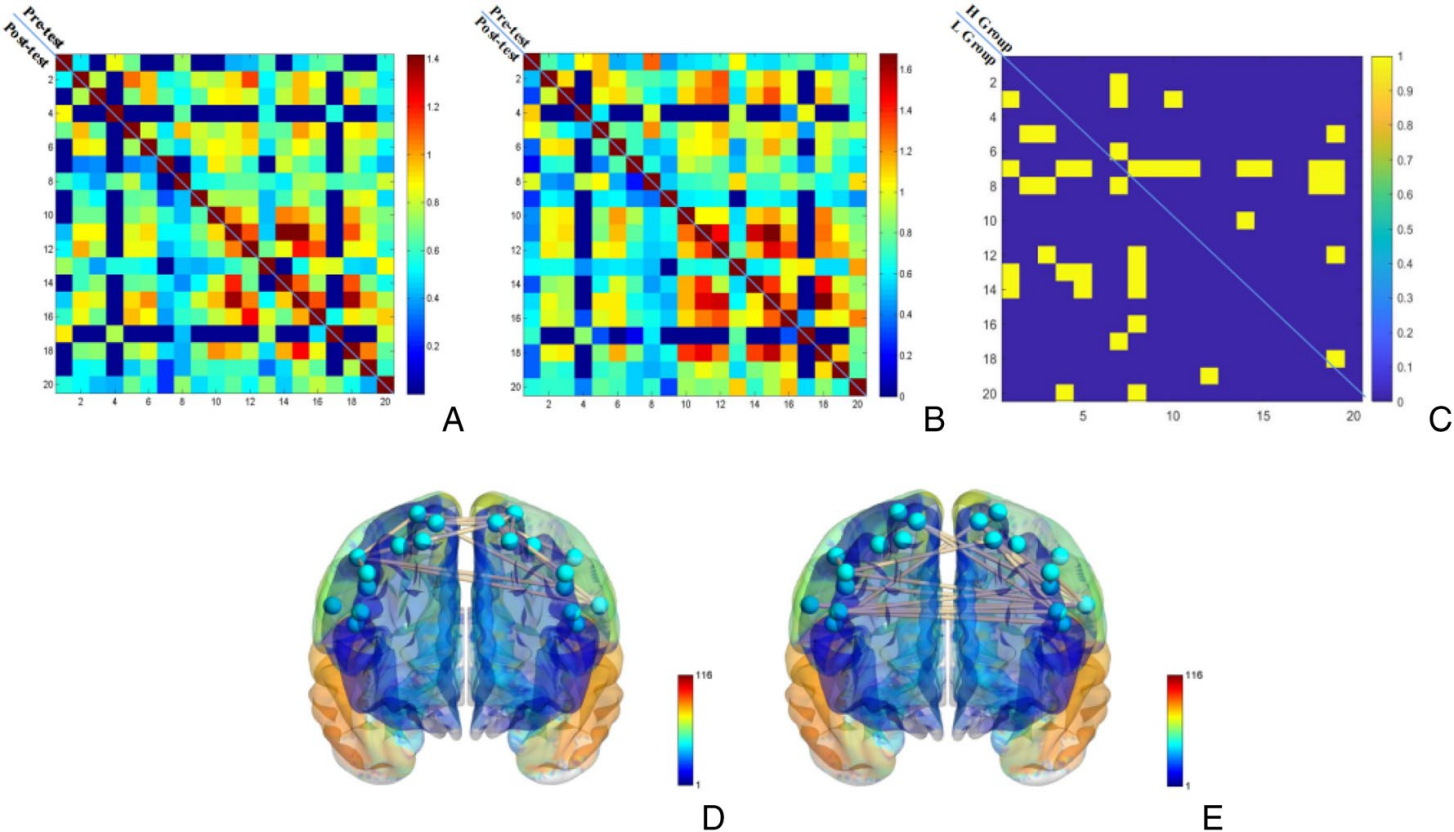
*p* value (*p* < 0.05, uncorrected) matrix map and brain network map of pre- and post-test of high-intensity AE in high and low fitness groups. *Note*: (**A**) and (**B**) show the RSFC matrices for the pre- and post-test for the high and low fitness groups, respectively, (**C**) shows the *p*-value (p < 0.05, uncorrected) matrices for the pre- and post-test for the high and low fitness groups, (**D**) and (**E**) show the brain networks with differential “edges” for the high and low fitness groups, respectively.

Paired samples *t*-tests were conducted on single edges of the RSFC pre- and post- high-intensity AE (n = 190) (Fig. 7), and the results showed that a total of 18 edges were significantly different pre- and post-test in the high fitness group, while a total of 23 edges in the low fitness group.It can be seen that at the high-intensity level, there is an inverse change in the number of single edges in the two groups. This suggests that the RSFC of the high fitness group was more stable than that of the low fitness group when stimulated with high-intensity load.

## Discussion

To investigate whether AE affects the RSFC in the motor cortex of college students, the present research applied the fNIRS technique to examine the changes in RSFC in the motor cortex of college students with different fitness levels at different exercise intensities. We compared the changes in RSFC of college students after AE for different exercise intensities and analyzed the moderating effect of fitness level. In the total RSFC, the results showed that the main effects of fitness level and time were significant, and that only the motor cortex RSFC of the high fitness group changed significantly after moderate-intensity AE, while the motor cortex RSFC of both groups did not change after high-intensity AE. This verifies the previous hypotheses: (1) different intensities of AE will have differential effects on motor cortex RSFC in college students and (2) fitness level has a moderating effect on the processing effects of AE.

On the other hand, the results for the number of difference “edges” showed the trends: at the moderate-intensity level, the number of difference edges was 23 for the high fitness group, while the number of difference edges was only 15 for the low fitness group. While at the high-intensity level, the high fitness group showed a decrease in the number of difference edges (18), but the low fitness group showed an increase in the number of difference edges (23). The results suggest that the RSFC of the motor cortex of the high fitness group was prone to adaptive changes (statistically significant changes) in response to moderate-intensity AE, but showed greater stability in response to high-intensity AE. However, the trend was diametrically opposed in the low motor group, which showed less significant changes in RSFC in response to moderate-intensity AE, but was more sensitive to higher intensity AE effects were more sensitive (but no statistically significant changes). So, why do exercise effects differ between different fitness levels groups, and what are the neural mechanisms involved in the emergence of these differences?

This research suggests that the RSFC of athletes may have the same characteristics as the “cardiac reserve”of the athlete’s heart: the athlete’s RSFC starts faster in response to general exercise tasks and has more space and stability when high-intensity tasks occur. The brain activity in resting state is a kind of "low running" process waiting for the execution of a task request, and this process is organized in specific functional networks, waiting to participate in the activation of a specific task^33^. Compared to the low fitness group, the high fitness group had lower total RSFC in resting state. However, after the exercise task started, RSFC quickly mobilized neural resources to cope with the needs of the exercise task, and the effect size was decreased (more stable) in the high-intensity exercise. This phenomenon may be due to the fact that the resting “RSFC reserve” was greater in the high fitness group, thus providing more flexible space for cognitive activity to be initiated^34,35^, and to meet the cognitive demands associated with different degrees of motor skill complexity^36^. In addition, the motor cortex is involved in the planning, execution and control of movement^37^. Athletes with higher levels of motor skill automation have fewer cognitive resources required to complete the planning, execution, and control of movement during exercise^38^. Thus, there would be a reduction in the effect size of in the effect of high-intensity exercise on RSFC in the present research. From the above evidence, it is clear that the different trends in effect size and number of individual variables between the two groups (high fitness group in line with the “inverted U hypothesis theory”, and low fitness group in line with the “drive theory” ) may be related to the differences in the “RSFC reserve”.

In addition, why is there a greater “RSFC reserve” in the motor cortex of people with high fitness,and what is the significance of a greater “RSFC reserve”? Some studies have shown that motor cortex plays a crucial role in regulating attention in the process of movement execution^39^, and the motor cortex is regarded as a key target brain area for evaluating the effect of neural rehabilitation strategies^40^. Some studies have proposed the concept of “mental effort”, that is, the tension generated when a certain amount of attention is devoted to the completion of a thinking task during controllable cognitive processing^41^. Theoretical models suggest that “mental effort” is needed to suppress the fatigue caused by physical movement in order to maintain a given physical movement task^42,43^. In the study, in order to hold the high or low intensity AE, both the high and low fitness groups needed to rely on “mental effort” to suppress fatigue produced by the body. If attentional focalization is not required during exercise, it is possible to conserve and preserve mental effort for the brain, ultimately resulting in longer duration of physical activity^44^. In this study, at high-intensity AE, a greater proportion of the post-exercise decrease in motor cortex RSFC occurred in the high fitness group, possibly because the high fitness group was more able to regulate attention and reduced the amount of “attentional focalization” in the motor cortex during exercise, thus saving more mental energy needed to hold the physical task, which in turn improves performance. This further suggests that the motor cortex of the college students with high group may have more “RSFC reserve”.

There is evidence from several studies that long-term physical activity can cause subtle structural changes in brain protein molecules and cells, such as an increase in brain-derived neurotrophic factor (BDNF) or a decrease in γ-aminobutyric acid type A (GABA-A) receptors^45–48^.This makes the nervous system more efficient and sensitive to changes in the internal or external environment (“plasticity”)^49–51^. Some studies indicated that the changes of brain neuronal plasticity and brain functional connectivity caused by physical activity are the basis for the improvement of cognitive function^52,53^. In the present research, only the total RSFC in the motor cortex of participants with high fitness changed significantly induced by moderate-intensity AE stimuli, which may be explained by the fact that participants with high fitness were more sensitive to AE stimuli and more prone to plasticity changes induced by AE compared to participants with low fitness. Tsai et al.^54^ found that only young people with higher fitness levels had reduced switching costs and enhanced neurophysiological benefits of AE, which contributed to better cognitive performance. Furthermore, Tsai et al.found in an earlier event-related potential (ERP) study that after AE, both high and low fitness groups were able to obtain response-related benefits such as reduced reaction time and increased area of correlated negative variation (CNV). However, only the high fitness group was able to obtain specific cognitive-processing benefits related to the allocation of attentional resources (e.g.,P3 amplitude) and cognitive preparation processes (e.g.,prefrontal CNV). Based on this, the present research suggests that the differences in the effects of AE on RSFC in people with high and low fitness in the experiment may be due to the moderating effect of fitness level on “AE-induced changes in cognitive performance levels and neurophysiological aspects of the brain”^55,56^.

In studies examining the effects of physical activity on cognitive function and brain health, another very important influence is exercise intensity, which is often manipulated as an independent variable during experiments^57–59^. Studies have shown that exercise intensity has an effect on cognitive behavioural performance and neurobiological signals, and that this effect has a “dose effect”^59–61^. There are two general theoretical models of the dose effect: the “drive theory” (linear relationship) and the “inverted U-shaped hypothesis”^62^. A study of the effects of AE on brain blood oxygenation in healthy young adults showed that after completing 30 min of aerobic exercise at three intensity levels (intensity of 52%, 68%, and 84%HRmax, respectively)at low,moderate,and high intensities, the most significant changes in oxygenated hemoglobin (HbO) levels in the prefrontal cortex were observed in the higher intensity exercise group, which concluded that increasing exercise intensity promotes cerebral oxygenation in the prefrontal cortex. Their findings are consistent with drive theory^63^. And their findings are consistent with drive theory^15^. In the present research, at the moderate-intensity level, the low fitness group showed a smaller number of different edges (15), while at the higher intensity level, an increase in the number of different edges (23) was observed. The results indicate that the trend in RSFC in the low fitness group is consistent with the drive theory hypothesis. However, the trend was reversed in the high fitness group: at the moderate-intensity level, the high fitness group showed a larger number of difference edges (23), while at the high-intensity level, a decrease in number of difference edges (18) was observed. This results indicate that the results for the high fitness group may be consistent with the “inverted *U*-shaped hypothesis”^16^. According to the inverted *U*-shaped hypothesis, this study suggests that neurophysiological changes and cognitive performance of college students at high fitness levels are best during moderate-intensity exercise, while high-intensity exercise may occur neural noise^64,65^ and decrease in exercise effects.

Our study only examined the effects of AE on RSFC in motor cortex at moderate- and high-intensity and did not examine the low intensity load. Although the results of the repeated measures ANOVA showed that the main effect of exercise intensity was not significant, exercise intensity is still an important variable that can cause changes in brain function. In the future, reliable physiological indicators such as blood lactate values^66,67^ can be used to set to finer intensity levels to obtain more data on exercise effects and further validate the inverted-*U* hypothesis theory or drive theory. The results of the study will have theoretical implications on how to design aerobic exercise programmes for people with different fitness levels during physical exercise. In addition, it will be necessary to include a non-exercise control condition in the future to exclude the influence of more irrelevant variables.

Some limitations should be considered when interpreting our findings: First, since the number of channels in the fNIRS device used in the experiment was only 20, the study only explored the effect of acute aerobic exercise on the RSFC of the motor cortex and failed to further analyze the relationship between the RSFC of the motor cortex and other brain regions. Therefore, future studies should apply multi-channel fNIRS equipment to monitor RSFC in multiple brain regions or the whole brain, to further verify the effects of AE (and long-term aerobic exercise) on the motor cortex and RSFC between the motor cortex and other functional brain regions.

Second, only moderate- and high-intensity exercise conditions were designed in this study, and other physiological and biochemical indexes were not further used to refine exercise intensity levels. Therefore, more physiological indicators should be included in future studies to refine exercise intensity, and non-exercise control condition should also be included to eliminate the interference of irrelevant variables. In addition, recent studies have found that low-intensity aerobic exercise can reduce the damage of sleep deprivation on the cognitive function of athletes^68^. In future studies, low-intensity aerobic exercise can be added to explore the impact of such exercise intensity on motor cortex RSFC of college students.

Third, since the study aims to test the moderating effect of fitness level on the effects of AE on RSFC in motor cortex, the above grouping methods on fitness level can meet the needs of the study purpose. However, in the future, more physiological indicators (i.e., in VO_2_peak values)can be introduced as grouping criteria in the future, and the study results may be more significant if the interference factors such as the type of exercise items are further separated.

Fourth, future studies could examine the characteristics of RSFC changes induced by different aerobic exercise modalities or exercise durations, as well as the correlation between RSFC changes and some specific cognitive and behavioral indicators, to justify the significance of our research: providing scientific guidance for young people with different fitness levels to choose scientific and reasonable exercise intensity.

Fifth, the sample size of our study was only determined based on past research experience^59,69^, which lacks scientific rigour. Similarly, for eliminating the interference effect of gender as an irrelevant variable, the research only recruited male subjects and had a lack of female subjects. So, a power analysis of the sample size is warranted in the future, and it is necessary to include female subjects with different fitness level in further studies to comprehensively investigate the complex relationship between exercise intensity and exercise effect in healthy college students.

Notably, in the future, it is worth considering the deeper and related physiological factors that influence exercise performance depending on exercise intensity and aerobic fitness, which could prompt further inquiry into functional connectivity between motor and cognitively salient (e.g., frontoparietal, dorsal attention, default mode) brain networks.

## Conclusion

In this study, we found a significant changes in resting-state functional connectivity (RSFC) in college students with high fitness at moderate-intensity acute aerobic exercise, whereas no significant changes at high-intensity AE. Fitness level has a moderating effect on the relationship between exercise intensity and RSFC, and people with high fitness are more susceptible to a greater range of changes in RSFC as a result of AE.

## Data Availability

The datasets used and/or analyzed during the current study available from the corresponding author on reasonable request.
